# A Case Series and Discussion on Surgical Treatment Strategy for Atypical Proximal Femoral Fractures Associated with Bisphosphonate Use

**DOI:** 10.7759/cureus.3670

**Published:** 2018-12-02

**Authors:** Brett Rocos, Thomas Fleming, Karen Harding, Mehool Acharya, Andrew Riddick, Mike Kelly

**Affiliations:** 1 Orthopaedics, North Bristol National Health Service Trust, Bristol, GBR; 2 Orthopaedic Surgery, North Bristol National Health Service Trust, Bristol, GBR

**Keywords:** trauma, geriatrics, resuscitation, blood transfusion

## Abstract

The aim of this study was to determine the incidence of atypical femoral fractures in our local population, study their current outcomes and present a novel surgical strategy based on these data. Patients who received surgical fixation of an atypical pattern proximal femoral fracture over a four-year period were identified and followed up in the clinic until union, revision surgery or death.

The local incidence of atypical femoral fractures is 1.1 per 1000 per annum amongst patients receiving bisphosphonates. Twelve fixation procedures were carried out in 10 patients. Intra-operative reduction and nailing led to an average deformity of 8.5° varus and 13° apex anterior. Five cases required revision surgery. Fifty percent of primary procedures resulted in radiographic union within two years. We suggest that the lateral side of the fracture should be considered a primary nonunion. We advocate undertaking a wedge excision to correct the bone to a valgus morphology and stabilising with an intramedullary nail and a lateral tension plate. Multicentre studies are needed to demonstrate the efficacy of any particular approach.

## Introduction

Bisphosphonates are widely used for the treatment of osteoporosis and have reduced the incidence of fragility fractures by up to 53% [1-2]. Since first identified by Odvina et al. in 2005, the association of long-term bisphosphonate use with atypical fractures of the proximal femur has become a concern [3-10], which led to the Medicines and Healthcare Products Regulatory Agency (MHRA) issuing safety updates in 2009 and 2011 regarding the prolonged use of bisphosphonates and the surveillance of patients taking the drugs over a long term [11]. Atypical femoral fractures are thought to occur due to bisphosphonates preventing the formation and action of osteoclasts, consequently preventing the normal remodelling of stress fractures with the effect of allowing propagation of the fracture line across the entire bone [6-7,12-13]. Histological analysis of the tissues taken from atypical femoral fractures has supported this theory by showing a paucity of osteoclasts within an immature new bone [14]. 

The diagnosis of atypical femoral fracture requires the injury to meet major and minor features. All major features must be satisfied, and although none of the minor features are required, they are strongly associated with the injury [11,15-16]. The incidence of bisphosphonate-associated fractures is thought to be approximately one per 1000 per annum, and the existing literature shows a high complication rate associated with treating these injuries, including nonunion, delayed union and metalwork failure requiring revision surgery [17-19]. The 2014 American Society of Bone and Mineral Research (ASBMR) task force identified the deficiencies in the management of these injuries and noted that no optimal surgical strategy has been described [15,20]. 

The purpose of this study was to determine the incidence of these fractures in our population, study the clinical and radiological outcomes in our cohort and present a novel surgical strategy based on these data.

## Materials and methods

A retrospective cohort methodology was employed. Patients who were treated for an atypical proximal femoral fracture in our unit between 1^st^ March 2009 and 31^st ^March 2013 were identified from a prospectively collected orthogeriatric database (non-proprietary), wherein details regarding the atypical fractures are collected. Patients were excluded if the fracture was distal to the isthmus or they failed to survive to discharge from the outpatient follow-up. Data were correlated with the Trauma and Orthopaedic admission and procedure database (Bluespier Patient Manager v.8.0S, Bluespier International, Droitwich, UK) to ensure no relevant cases were absent from the analysis. The patients' clinical records and digitally stored investigations were reviewed to confirm the accuracy of the diagnosis and the treatment data. Pre-operative radiographs were assessed in the orthopaedic multidisciplinary meeting to confirm the diagnosis of atypical proximal femoral fracture. Post-operative radiographs were reviewed in the same multidisciplinary meeting to determine the accuracy of reduction, type of fixation and any augmentation used. An independent consultant orthopaedic trauma surgeon who was blinded to the patients' medical and pharmacological history then subsequently reviewed each radiograph to confirm both the diagnosis and treatment characteristics.

Follow-up radiographs and clinical records were reviewed to determine progression to medial and lateral union. Union was defined by the presence of callus bridging the two fragments visible on orthogonal radiographs as assessed by both the consulting clinician and an independent, blinded consultant trauma surgeon. No clinical criteria for the union were set, although the absence of symptoms supported union when identified radiologically. Any failure of fixation or revision surgery was also noted. 

## Results

Within the catchment area of our unit, the latest data available (2012) show that 4490 patients had a diagnosis of osteoporosis, of which 3395 underwent bisphosphonate therapy [21]. This gives rise to an expected annual incidence of atypical femoral fractures of 3.3 fractures per year presenting to the trauma service. 

Seventeen primary operations for atypical femoral fractures were carried out for 15 patients between 1^st^ March 2009 and 31^st^ March 2013, representing 1% of 1831 proximal femoral reconstructive operations performed during that period in our tertiary trauma unit. The calculated incidence of atypical femoral fractures in our region is 1.1 per 1000 per annum amongst patients taking bisphosphonates. This is comparable to the established incidence of 1:1000 per year published by Schilcher et al. in 2009 [17].

Three procedures were excluded due to the fracture being distal to the isthmus. Two were excluded as they were prophylactic procedures for the impending fractures. The 12 remaining procedures were carried out in 10 patients. Nine patients were female with a mean age of 71 years (Table 1).

**Table 1:** The demographics, clinical details and surgical results of patients with atypical proximal femoral fractures treated in our unit between March 1, 2009 and March 31, 2013.

Case	Sex	Age	Fixation device	Duration of prodromal pain (weeks)	Position of final reduction	Time to medial union (days)	Time to lateral union (days)	Difference in time to union (days)	Revision procedure	Drug	Length of drug treatment	Co-morbidities
1	F	64	Gamma 3 (Stryker)	None recorded	Varus 13°	332	Nonunion	-	None	Alendronate	Unknown	Renal transplant, Type 2 diabetes
2	F	67	Gamma 3 (Stryker)	4	Varus 13°	89	Nonunion	-	None	Intravenous ibandronate, Alendronate Prednisolone	3 years	Breast carcinoma, colitis, emphysema
2 (2^nd^ fracture)	F	71	Gamma 3 (Stryker)	None recorded	Varus 14° flexion 20°	82	82	0	None	Intravenous ibandronate, Alendronate Prednisolone	>5 years	Breast carcinoma, colitis, emphysema
3	F	81	Gamma 3 (Stryker)	None recorded	Varus 11° flexion 7°	342	524	182	None	Alendronate	Unknown	Ankylosing spondylitis, Osteoporosis
4	F	73	Gamma 3 (Stryker)	None recorded	Neutral	117	236	119	Revised at five days for iatrogenic fracture to plate and revision nailing	Alendronate Prednisolone	4 years	Polymyalgia rheumatica
5	F	64	Gamma 3 (Stryker)	2	Varus 8° flexion 11°	none	Nonunion	-	Revision to blade plate at 14 months for nonunion. Fully united four months post revision	Alendronate	1 month	Chronic obstructive pulmonary disease, obstructive sleep apnoea
5 (2^nd^ fracture)	F	66	Gamma 3 (Stryker) & anterior plate	None recorded	Varus 4°	181	Nonunion	-	Revised at 10 months for broken nail	Alendronate	2 years	Obesity, chronic obstructive pulmonary disease, obstructive sleep apnoea, ischaemic heart disease
6	M	66	Gamma 3 (Stryker) & cable	1	Varus 10 °	76	Nonunion	-	Revised at 15 months to blade plate for broken nail	Alendronate	16 months	Rheumatoid arthritis
7	F	79	Gamma 3 (Stryker)	None recorded	Varus 3°	66	192	126	None	Alendronate	4 years	Polymyalgia rheumatica
8	F	74	Gamma 3 (Stryker)	26	Varus 15°	86	373	287	None	Alendronate	8 years	Osteoporosis
9	F	67	Gamma 3 (Stryker)	3	Varus 7°	none	Nonunion	-	Revised at four months to revision nail and lateral tension band plate for broken nail. Fully united at six Months	Alendronate	5 years	Polymyalgia rheumatica
10	F	75	T2 recon nail (Stryker)	None recorded	Varus 4°	76	361	285	None	Alendronate	6 months	Rheumatoid arthritis, lumbar stenosis, gout

Seven cases required an open approach to achieve a reduction. Anatomical reduction was achieved in each case, giving rise to a femur with an average of 8.5° varus deformity. Three patients showed an average apex anterior malreduction of 13° visible on the lateral radiograph. 

Fifty percent of the primary procedures resulted in radiographic evidence of both medial and lateral union within a two-year follow-up. In those patients who united both medially and laterally, the lateral union took an average of 167 days longer than the medial side (range: 0-287 days). 

Five revision procedures were required: one for nonunion, three for the failure of metalwork and a single case for iatrogenic periprosthetic fracture occurring at the primary procedure. The details of revision surgery can be seen in Table 1.

## Discussion

The literature describes the prevalence of atypical femoral fractures as 0.5-1:1000 per annum, a figure confirmed in our own series [17,22-24]. It is an uncommon injury and each trauma service will see only a few cases every year. The present literature suggests managing these injuries with anatomical reduction and cephalo-medullary nail fixation [25]. Our results echo those of a similar case series, showing a low rate of union and a high re-operation rate with this treatment strategy [26-29]. This group of patients tends to have multiple co-morbidities (the median American Society of Anesthesiologists (ASA) Physical Status grade in this study was three). The development of a surgical strategy that reduces the reoperation rate would, therefore, be desirable.

Based on the data from this study, we hypothesize that the femur deforms into varus because of the inhibited physiology leading to the propagation of the lateral cortical stress responses until a fracture results. This then fails to unite due to the continued action of bisphosphonates and the adjacent cortical reaction producing the typical beaking [20,30]. This is demonstrated by the femora in this cohort showing a mean varus deformity of 8.5° at the time of fracture. The non-united stress fractures continue to be subjected to a varus moment causing their propagation through the remaining bone to complete the femoral fracture. 

We suggest that the lateral side of the atypical proximal femoral fractures should be considered a primary nonunion and dealt with as such [11,18]. By contrast, the medial cortical break behaves like an acute fracture, with haematoma and intramembranous ossification. This suggests that this lateral portion of the fracture represents a chronic, sclerotic, nonunion with poor healing potential. The fracture propagates to completion when the remaining intact bone becomes insufficient to support the patient’s weight [6-7,12-13]. Furthermore, it is likely that the cortical defect propagates over a long period (increasing varus appearance radiographically) prior to eventual fracture completion. This is reflected in that half of our cohort who complained of prodromal thigh pain for up to 18 months.

The 42% re-operation rate in our series shows that the currently accepted fixation techniques are inadequate. At best, they are only able to correct alignment back to the pre-existing varus deformity present just prior to fracture completion. This does not address the poor mechanical environment, particularly in the face of impaired biology meaning that the lateral healing is at best very delayed putting the implant at a high risk of failure before union occurs. 

**Figure 1:**
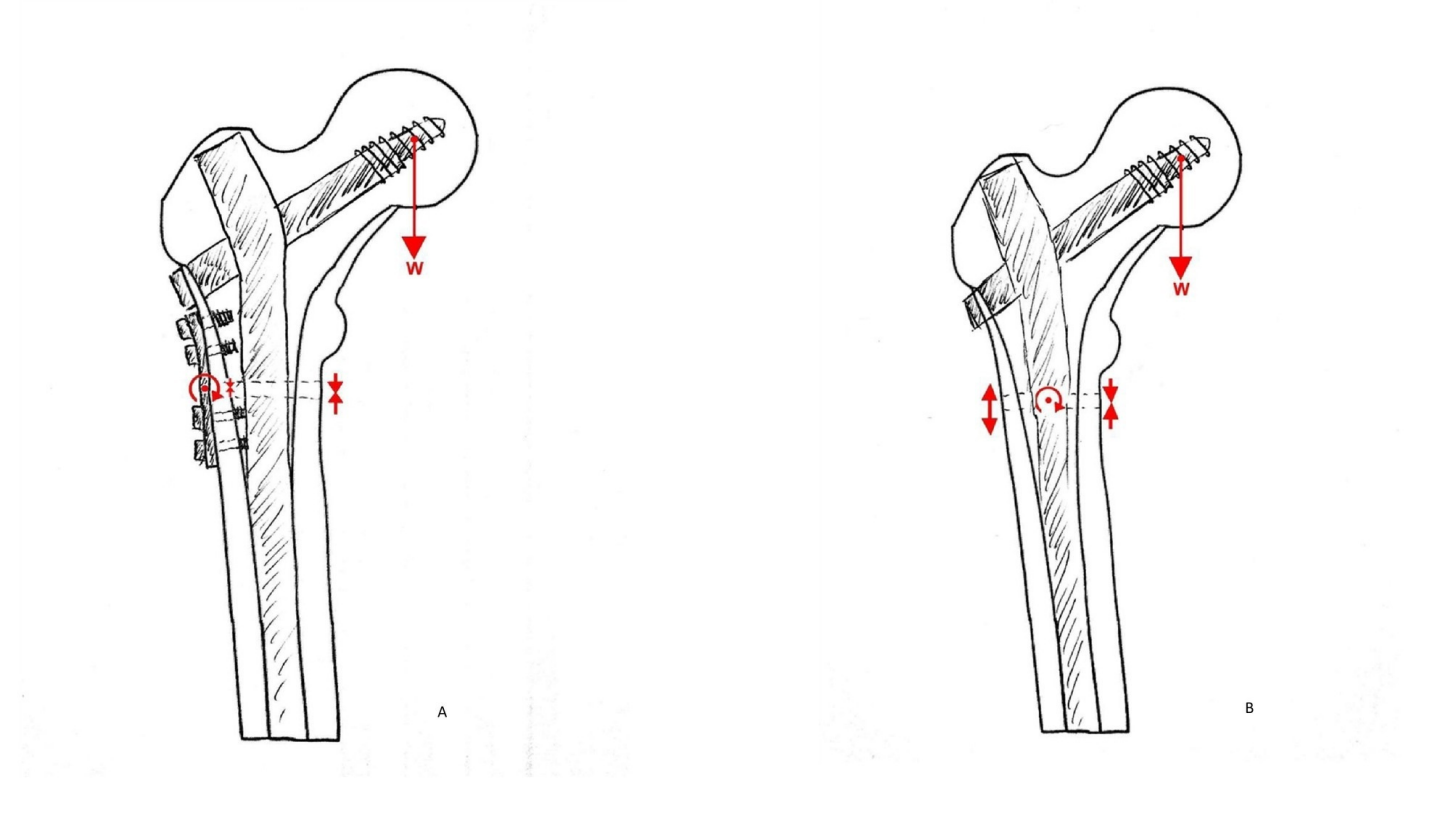
Forces applied across an atypical fracture following traditional and novel fixation techniques The red arrows indicate the direction of force applied by the body weight (w) and the resultant forces across the fracture caused by the fixation technique. A) Cephalomedullary nail alone caused distraction at the lateral side of the fracture, increased if the natural varus is not corrected. B) With the addition of a lateral tension band plate, wedge excision of the lateral cortex and valgising reduction, compression is achieved across the entire fracture.

We advocate a surgical strategy that anticipates the impaired biology and addresses the inadequate mechanical environment. This is achieved by undertaking a wedge excision to remove the sclerotic lateral margin and correcting the bone to a valgus morphology. Stabilization is then achieved using an intramedullary nail and lateral tension plate positioned just posterior to the nail on the lateral view at the level of the fracture (Figure 1). This strategy optimizes the mechanical environment creating a situation where the mechanics of the fracture are in optimal circumstances for healing. This strategy will now be adopted in our unit as a standard approach to this injury and results will be reported on when available. 

## Conclusions

Atypical femoral fractures are uncommon and difficult to manage reproducibly. Current techniques have a 30-40% incidence of re-operation. We therefore now advocate of an initial surgical management strategy that uses a valgising wedge osteotomy to correct the evolved varus morphology and improve the mechanics of the final construct. As this is a rare injury, multicentre studies are needed to demonstrate the efficacy of any particular approach.
